# Prevalence, antimicrobial susceptibility patterns, serotypes and risk factors for group B streptococcus rectovaginal isolates among pregnant women at Kenyatta National Hospital, Kenya; a cross-sectional study

**DOI:** 10.1186/s12879-020-05035-1

**Published:** 2020-04-22

**Authors:** Salano Clayton Jisuvei, Alfred Osoti, Maina Anne Njeri

**Affiliations:** 1 Institute of Tropical and Infectious Diseases, University of Nairobi, Nairobi, Kenya; 2 Research and Programs Department, Kenyatta National Hospital, Nairobi, Kenya; 3 Department of Obstetrics and Gynaecology, University of Nairobi, Nairobi, Kenya; 4 Department of Medical Microbiology, University of Nairobi, Nairobi, Kenya

**Keywords:** Group B Streptococcus, Pregnant women, Antimicrobial susceptibility, Serotypes, Kenya

## Abstract

**Background:**

Estimates of Group B Streptococcus **(**GBS) disease burden, antimicrobial susceptibility, and serotypes in pregnant women are limited for many resource-limited countries including Kenya. These data are required to inform recommendations for prophylaxis and treatment of infections due to GBS.

**Methods:**

We evaluated the prevalence, antimicrobial susceptibility patterns, serotypes, and risk factors associated with rectovaginal GBS colonization among pregnant women receiving antenatal care at Kenyatta National Hospital (KNH) between August and November 2017. Consenting pregnant women between 12 and 40 weeks of gestation were enrolled. Interview-administered questionnaires were used to assess risk factors associated with GBS colonization. An anorectal swab and a lower vaginal swab were collected and cultured on Granada agar for GBS isolation. Positive colonies were tested for antimicrobial susceptibility to penicillin G, ampicillin, vancomycin, and clindamycin using the disk diffusion method. Serotyping was performed by latex agglutination. Logistic regression was used to identify factors associated with GBS colonization.

**Results:**

A total of 292 women were enrolled. Median age was 30 years (Interquartile range {IQR} 26–35) and a median gestational age of 35 weeks (IQR 30–37). Overall GBS was identified in 60/292 (20.5%) of participants. Among the positive isolates, resistance was detected for penicillin G in 42/58 (72.4%) isolates, ampicillin in 32/58 (55.2%) isolates, clindamycin in 14/46 (30.4%) isolates, and vancomycin in 14/58 (24.1%) isolates. All ten GBS serotypes were isolated, and 37/53 (69.8%) of GBS positive participants were colonized by more than one serotype. None of the risk factors was associated with GBS colonization.

**Conclusion:**

The prevalence of GBS colonization was high among antenatal women at KNH. In addition, a high proportion of GBS isolates were resistant to commonly prescribed intrapartum antibiotics. Hence, other measures like GBS vaccination is a potentially useful approaches to GBS prevention and control in this population. Screening of pregnant mothers for GBS colonization should be introduced and antimicrobial susceptibility test performed on GBS positive samples to guide antibiotic prophylaxis.

## Background

*Streptococcus agalactiae* also called Group B Streptococcus (GBS), causes invasive infections mainly in pregnant women and neonates [1]. Temporary GBS colonization of the female urogenital tract is a known causative factor of neonatal infections acquired during the childbirth process [2]. Acquisition of GBS can be perinatal during labour or *inutero* through transmission of the bacteria from the maternal vaginal or anorectally colonized mucosa. GBS is also a risk factor for neonatal sepsis and mortality that is associated with preterm birth [3]. Neonatal GBS infection can be early or late onset with early-onset disease (EOD) ensuing within the first 7 days of life and presents as pneumonia, meningitis, and sepsis that carries a mortality rate of up to 20% [2].

Global rates of GBS colonization vary widely [4] with high prevalence rates of between 15 and 33% being reported in the United States, Jordan and Gambia [5] while low prevalence rates of less than 10% have been reported in Italy, Turkey and Iran [6]. Though excellent data on GBS exists among developed countries, there is paucity of information on the recto-vaginal colonization and associated maternal and neonatal complications due to GBS as well as serotypes in circulation, among many resource limited countries [5, 7]. Among the few GBS studies conducted in sub-Sahara Africa, high colonization rates of 23 and 16.5% have been reported in Tanzania and Malawi respectively [7]. In the most recent study in Kenya, Seale, Koech [8] reported a GBS prevalence of 12% among expectant mothers in Kilifi county while an earlier study by Salat et al done in 2009, found a high prevalence of 25.2% among pregnant women at KNH [9]. Even so, these two studies were limited in their scope to the prevalence and risk factors of GBS colonization but failed to investigate the antimicrobial susceptibility or serotypes of isolated GBS in their populations.

Neonatal sepsis contributes to 7% of neonatal mortality deaths in Kenya [10] with *Escherichia coli* as the main associated causative agents [11]. The contribution of GBS on causation of neonatal sepsis in Kenya has received little focus; this is despite the overwhelming evidence from developed countries that has associated rectovaginal GBS colonization with EOD [12]. Unpublished hospital statistics from KNH for the period January 2015 to December 2015 ranked neonatal sepsis as the highest infectious cause of neonatal death at 20%. Based on the year 2015 KNH statistics and an estimated 25.2% GBS prevalence, approximately 500 neonates die annually at the facility translating to approximately two deaths daily from early onset GBS disease [13]. The GBS related infant mortality may even be higher in lower resource facilities in the country.

In Kenya, pregnant women are not routinely screened for rectovaginal GBS colonization [14]. As such, there is paucity of data on the prevalence, antimicrobial susceptibility pattern, serotypes and risk factors of rectovaginal GBS colonization in Kenya. This study sought to contribute to this knowledge gab by determining the prevalence, serotypes, antimicrobial susceptibility patterns and risk factors to GBS rectovaginal colonization among pregnant women in this population.

## Methods

This was a cross sectional study conducted between August and November 2017. A population of pregnant women between the gestational ages of 12 to 40 weeks receiving antenatal care (ANC) at KNH was used. The study was conducted at KNH ANC clinic. KNH is the largest national reference hospital in Kenya. The ANC clinic attends to an average of 120 women each clinic day [13].

### Inclusion criteria

Pregnant women without pregnancy complications such as placenta previa and vaginal bleeding and those above 18 years were included in the study. Women with current history of per vaginal bleeding, ruptured membranes, in labour and those having been on antibiotics treatment within 2 weeks prior to the study were excluded.

### Data collection instruments

Interviewer-administered questionnaires were used to gather obstetric and other study relevant information. Laboratory samples collected were two swabs, one from the lower vagina and the other from the anorectal area. Sterile Dacron swabs were used in collecting the samples.

In summary: the participant lay on the examination couch in a semi-lithotomy position and without speculum placement, the vagina labia were parted and the lower vagina swabbed using a sterile Dacron swab, immediately after, the swab was inoculated on an appropriately labelled Group B Streptococcus selective agar (GBSA/granada) plate (Hardy diagnostic). With the second swab, the anorectal area was swabbed and immediately inoculated on an appropriately labelled GBSA plate. The plate was placed in a cooler box and transported to the laboratory within 6 h of sample collection.

#### GBS isolation

In the lab, plates were streaked and incubated anaerobically at 35 ± 2 °C for 18–24 h as per the GBSA medium manufactures’ instruction. After 24 h, the plates were inspected for growth. GBS positive cultures were identified by the characteristic orange coloured colonies on GBSA agar. Gram stain was used to confirm the orange colonies as streptococcus.

A portion of GBS positive colonies was picked and stored at − 20 °C in skimmed milk until testing for GBS serotypes.

Another portion of GBS positive colonies was picked and emulsified in a clean test tube containing sterile normal saline for antimicrobial susceptibility testing.

#### Antimicrobial susceptibility testing

Antimicrobial susceptibility profile of isolated GBS was tested using disk diffusion test. The turbidity of the emulsified colonies was standardised to 0.5 McFarland and inoculated on Muller Hinton Agar enriched with 5% sheep blood.

Penicillin G-10 μg (Hardy diagnostic, Lot: 385950), ampicillin-10 μg (Hardy diagnostic, Lot: 391375), clindamycin-2 μg (Hardy diagnostic, Lot: 384766) and vancomycin-30 μg (Hardy diagnostic, Lot: 388310) drug discs were placed on the surface of the plate equidistant from each other and the plates incubated anaerobically at 35 ± 2 °C for 18–24 h. The zones of inhibition as set by Clinical and Laboratory Standards Institute (CLSI) 2011 (penicillin G-10 units {S ≥ 24 mm}, ampicillin-10 μg {S ≥ 24 mm}, clindamycin-2 μg {R ≤ 15 mm, S ≥ 19 mm} and vancomycin30μg {S ≥ 17 mm}) were used to determine the susceptibility of GBS to the tested drugs. CLSI 2011 guidelines [15] were used in performing this test. The antibiotics used in the susceptibility tests are those used for intrapartum management of GBS at KNH [13].

#### Serotyping

Serotyping was determined by latex agglutination test using Immulex Strep-B kit (Statens serum Institute-Denmark Article 54,991/Lot: V3). For each GBS isolate, on a carbon test card with 10 circles labelled corresponding to the each GBS serotypes, a 10 μl drop containing GBS colonies emulsified in normal saline was placed. A 10 μl drop of the corresponding Immulex Strep B antisera was added to the suspension and mixed before gently rocking while observing for agglutination. This test was done according to the manufacturer’s instruction.

### Data analysis

Collected data was analysed using STATA® version 13. Participant characteristics were analysed descriptively, Mann Whitney U test and Chi-square were used to compare characteristics between colonized and uncolonized women while logistic regression was used to identify factors associated with GBS colonization. *P* values ≤0.05 were taken to be statistically significant.

## Results

Between August and November 2017, a total of 350 pregnant women were screened, 292 eligible pregnant women between 12 and 40 weeks gestation consented and were enrolled to participate in the study. From these, 292 questionnaires were fully filled and 292 (100%) vaginal swab samples and 288 (98.6%) anorectal swab samples collected for GBS culture. Four participants declined to have anorectal swabs collected.

### Socio-demographic characteristics

The median age of participants was 30 years (IQR 26–35). About one in five (*n* = 55, 18.8%) were aged below 25 years, majority (*n* = 177, 60.6%) were aged between 26 to 35 years while another one in five were aged 36 years or older (*n* = 60, 20.5%). The median gestation age of study participants was 35 weeks (IQR 30–37). Less than half (*n* = 132, 45.2%) of the participants were of gestation age 34 weeks and below while 15.1% (*n* = 44) were term i.e. greater than 37 gestation weeks (Table 1).

**Table 1:** Sociodemographic and obstetric characteristics of study participants

**Participant characteristics**	**Total (*N*** **= 292) (100%)**
Age (Years)	
≤ 25	55 (18.8%)
26–35	177 (60.6%)
≥ 36	60 (20.5%)
Gestation age (weeks)	
< 35	132 (45.2%)
35–37	166 (39.7%)
> 37	44 (15.1%)
Parity	
0	53 (18.2%)
1	83 (28.4%)
> 1	156 (53.4%)

### Obstetric and clinical characteristics

Half of the participants (*n* = 156, 53.4%) were multiparous with a median parity of three pregnancies (IQR 2–4). Only 53 (18.2%) of the pregnant women were primigravida. Approximately three quarters of participants (*n* = 210, 71.9%) had one or more live births, however, 29 (9.9%) of participants though having been pregnant before, had never delivered a live birth (Table 1).

### Maternal GBS colonization

Out of the 292 study participants, 20.5% (*n* = 60) tested positive to GBS recto-vaginal colonization on both culture and gram stain. GBS was cultured from vaginal swabs of 16.8% (49/292) participants while anorectal swabs of 18.8% (55/288) participants were GBS positive. On the other hand, 15.1% (44/292) of study participants had both rectal and vaginal GBS colonization.

### Sociodemographic and obstetric characteristics of GBS rectovaginal colonization among pregnant women receiving antenatal care at Kenyatta National Hospital

The prevalence of GBS was highest among participants within the 26–35 years age bracket at 13.0% (*n* = 38), approximately one in twenty participants (*n* = 12, 4.1%) aged above 36 years were GBS colonized. Participants within the 35–37 weeks gestation bracket had the highest prevalence of GBS (*n* = 28/292, 9.6%) followed by participants of gestational age below 35 weeks (*n* = 26/292, 8.9%). The prevalence of GBS was lowest among participants of gestational age above 37 weeks (*n* = 8/292, 2.1%).

The prevalence of GBS among participants with a history of stillbirth was 6.5% (*n* = 19), abortion or ectopic pregnancy was 3.1% (*n* = 9) while among those with history of preterm birth it was 4.1% (*n* = 12); for participants with a history of fore water break, more than 18 h before labour, the prevalence was 3.4% (*n* = 10). The prevalence was 2.1% (*n* = 6) among participants allergic to penicillin, and 1.0% (*n* = 3) among HIV-positive participants; participants with white coloured vaginal discharge had the highest GBS colonization with a prevalence of 11.0% (*n* = 32) followed by clear coloured vaginal discharge with a prevalence of 4.5% (*n* = 13) (Table 2).

**Table 2:** Sociodemographic and obstetric characteristics of GBS colonized women

**Sociodemographic and obstetric characteristics**	**GBS culture results**		
	**Negative (*N*** **= 232)**	**Positive (*N*** **= 60)**	***P***
	**n (%)**	**n (%)**	
Maternal age (Years)			
< 26	45 (15.4%)	10 (3.4%)	0.864
26–35	139 (47.6%)	38 (13.0%)	
> 36	48 (16.4%)	12 (4.1%)	
Gestation age (weeks)			
< 35	106 (36.3%)	26 (8.9%)	0.323
35–37	88 (30.1%)	28 (9.6%)	
> 37	38 (13.0%)	6 (2.1%)	
Parity			
0	43 (14.7%)	10 (3.4%)	0.499
1	69 (23.6%)	14 (4.8%)	
> 1	120 (41.1%)	36 (12.3%)	
Number of prior live births if parity > 0			
0	25 (8.6%)	4 (1.4%)	0.566
> 0	164 (56.2%)	46 (15.8%)	
Number of prior stillbirths	74 (25.3%)	19 (6.5%)	0.935
History of pregnancy loss in prior pregnancies (abortion or ectopic)	23 (7.9%)	9 (3.1%)	0.308
History of preterm birth in prior pregnancy	45 (15.4%)	12 (4.1%)	0.945
Rupture of membranes for > 18 h before labour in prior pregnancy	48 (16.4%)	10 (3.4%)	0.688
Past history of neonatal death in first week of birth	23 (7.9%)	9 (3.1%)	0.526
Past history of neonatal infection after birth	25 (8.6%)	5 (1.7%)	0.782
Fore water break in past pregnancy Rupture of membranes?	5 (1.7%)	2 (.7%)	0.595
Allergy to penicillin			
Yes	9 (3.1%)	6 (2.1%)	0.051
Don’t Know	50 (17.1%)	7 (2.4%)	
HIV status			
Positive	12 (4.1%)	3 (1.0%)	0.628
Colour of vaginal discharge			
Yellow	25 (8.6%)	7 (2.4%)	0.942
Brown	32 (11.0%)	8 (2.7%)	
White	132 (45.2%)	32 (11.0%)	
Clear	43 (14.7%)	13 (4.5%)	

*GBS* Group B Streptococcus, n number of participants within group, % percentage of participants within group, Chi-square statistics was used test statistical; difference between GBS colonized and uncolonized participants

### Antimicrobial susceptibility pattern

Penicillin G, ampicillin and vancomycin susceptibility pattern was tested on 58 samples while clindamycin susceptibility was tested on 46 samples. GBS bacterium isolated in this population had the highest resistance to penicillin G (72.4%, *n* = 42/58) and ampicillin (55.2%, *n* = 32/58). Resistance to vancomycin and clindamycin was at 24.1% (*n* = 14/58) and 30.4% (*n* = 14/46) respectively. In general, a slightly higher percentage of GBS isolates from the anorectal canal was resistant to tested antimicrobials in comparison to those isolated from the lower vagina. However, the difference between the two proportions was not statistically significant (Table 3).

**Table 3:** Difference between proportions of antimicrobial resistant GBS isolated from the lower vagina and those isolated from the anorectal canal

**Antimicrobial susceptibility**		**GBS colonization**		**P**
		**Vaginal Positive**	**Anorectal Positive**	
		**(n, %)**	**(n, %)**	
Penicillin	Susceptible	15 (31%)	13 (25%)	0.499
	Resistant	34 (69%)	40 (75%)	
Ampicillins	Susceptible	24 (49%)	23 (43%)	0.544
	Resistant	25 (51%)	30 (57%)	
Vancomycin	Susceptible	39 (80%)	40 (75%)	0.546
	Resistant	10 (20%)	13 (25%)	
Clindamycin	Susceptible	28 (70%)	29 (69%)	0.922
	Resistant	12 (30%)	13 (31%)	

GBS Group B Streptococcus, n Number of positive GBS isolates, % percentage of isolates within group, *P* value obtained by comparing proportion of antimicrobial resistant isolated from anal and rectal canal

### Serotypes of isolated group B Streptococcus bacteria and their antimicrobial pattern

Out of the 60 GBS positive samples, serotype testing was conducted on 53 samples; seven samples did not grow after repeated sub-culturing. All ten known GBS serotypes occurred in this population. Serotype Ia was the most common serotype isolated from 75.9% of cultures followed by serotype III at 62.0%. Serotype IV was the least occurring serotype at 36.0%.

There was high resistance to penicillin G and ampicillin by all isolated GBS serotypes that ranged from 68 to 84% and 57 to 78% respectively. In comparison, resistance to vancomycin and clindamycin was low ranging between 16 to 26% and 15 to 33% respectively.

Serotypes IV isolates were the most resistant to penicillin G and ampicillin at 83.3 and 77.8% respectively while serotype Ia had the highest resistance to clindamycin at 33.3%; serotype VI was the most resistant to vancomycin at 25.9%. Overall, serotype IV was the most resistant to all tested antimicrobials while serotype II was the least resistance to tested antimicrobials.

### Association between GBS serotype and antimicrobial susceptibility among pregnant women receiving antenatal care at Kenyatta National Hospital

As summarized in Table 4, being colonized with serotype Ia was associated with penicillin resistance (OR 4.8; CI: 1.265–18.311, *p* = 0.021). Colonization with serotypes III (OR 6.84; CI:1.899–24.672, *p* = 0.003), IV (OR 5.12; CI:1.372–19.077, *p* = 0.015), VI (OR 4.45; CI:1.353–14.653, *p* = 0.014), and VIII (OR 4.67; CI:1.255–17.358, *p* = 0.022) was associated with ampicillin resistance. None of the isolates was associated with either vancomycin or clindamycin resistance.

**Table 4:** Association between GBS serotype and antimicrobial pattern indicating OR

**Serotype**	**Penicillin G**			**Ampicillin**			**Vancomycin**			**Clindamycin**		
	**OR**	**95% CI**	**P**	**OR**	**95% CI**	**P**	**OR**	**95% CI**	**P**	**OR**	**95% CI**	**P**
**Ia**	4.81	**1.265–18.311**	**0.021**	3.52	0.926–13.353	0.065	0.81	0.180–3.635	0.781	1.33	0.294–0.709	0.71
**Ib**	1.25	0.343–4.558	0.735	2.41	0.684–8.471	0.171	0.82	0.198–3.432	0.79	1.38	0.312–0.668	0.67
**II**	1.08	0.321–3.626	0.902	2.73	0.862–8.625	0.088	0.54	0.137–2.157	0.385	0.33	0.071–0.154	0.15
**III**	1.68	0.490–5.745	0.41	6.84	**1.899–24.672**	**0.003**	1.86	0.426–8.087	0.411	1.31	0.307–0.714	0.71
**IV**	3	0.717–12.553	0.132	5.12	**1.372–19.077**	**0.015**	1.02	0.254–4.104	0.977	0.74	0.158–0.702	0.7
**V**	1.17	0.346–3.933	0.804	1.33	0.434–4.094	0.615	1.5	0.377–5.965	0.565	0.76	0.182–0.714	0.71
**VI**	3.38	0.947–12.098	0.061	4.45	**1.353–14.653**	**0.014**	1.66	0.418–6.606	0.47	1.5	0.351–0.585	0.59
**VII**	1.89	0.519–6.868	0.334	3.48	0.99–12.242	0.052	0.83	0.202–3.435	0.801	0.29	0.062–0.124	0.12
**VIII**	2.25	0.567–8.927	0.249	4.67	**1.255–17.358**	**0.022**	1.33	0.286–6.214	0.714	0.76	0.149–0.746	0.75
**IX**	2.25	0.478–10.595	0.305	3	0.676–13.309	0.148	0.85	0.141–5.070	0.855	0.48	0.087–0.399	0.4

*GBS* group b streptococcus, *OR* odds ratio, *CI* confidence interval, Number in bold are statistically significant *P* < 0.05

### Risk factors for group B Streptococcus colonization among pregnant women receiving antenatal care at Kenyatta National Hospital

Logistic regression was used to determine predictors of positive GBS colonization. The variables were analysed both individually and in combination to ascertain their ability to predict positive GBS colonization. None of the variables in this study was a predictor of positive GBS colonization as indicated by the greater than 0.05 *p* value in Table 5.

**Table 5:** Risk factors of GBS among study participants

**GBS Results**	**Odds Ratio**	**95% Conf. Interval**	***P***
Maternal age (Years)	1.0	0.94–1.06	0.868
Parity	1.1	0.77–1.51	0.656
Parity group	2.3	0.87–5.99	0.092
Gestation Age	1.0	0.93–1.11	0.710
Gestation groups	0.8	0.45–1.51	0.528
Number of prior live births	1.1	0.77–1.51	0.669
Still births	0.7	0.46–1.16	0.187
History of pregnancy loss in prior pregnancies (abortion or ectopic)	1.3	0.77–2.20	0.331
History of preterm birth in prior pregnancy	1.0	0.64–1.51	0.948
Rupture of membranes for > 18 h before labour in prior pregnancy	0.7	0.30–1.60	0.396
Past history of neonatal death in first week of birth	2.1	0.80–5.60	0.130
Neonatal infection	0.5	0.15–1.61	0.238
Fore water break in past pregnancy/Rupture of membranes	1.4	0.23–8.41	0.725

*GBS* Group B Streptococcus, Chi-square statistics was used to obtain *P* value

## Discussion

At 20.5%, the rectovaginal prevalence of GBS among pregnant women receiving antenatal care at KNH was slightly higher than that reported by previous studies in this region or similar settings. This is comparable to findings in Mombasa, Kenya in which Cools, Jespers [16] reported a GBS prevalence of 20.2%. The prevalence of GBS has been reported to vary between 10 and 35% in the USA [4] and 1 and 30% in other developing countries [4, 16–18]. Even so, this prevalence is slightly lower than that reported by Salat et al, in a study conducted in the same setting in Kenya [9]. Difference of the prevalence in the two studies could be explained by the difference in gestational ages of women who formed study participants of the two studies. Salat restricted his participants to women of gestational age 35 to 37 weeks while this study included participants from gestational ages 12 to 40 weeks. Previous studies [19] have reported gestational age of women as a risk factor to GBS with the prevalence being higher among women of gestational age 35 to 37 weeks [5, 19]. Our study is consistent with this observation as grouping of participants based on gestational age, found a higher GBS prevalence of 9.6% among those in the gestational age 35 to 37 in comparison to other gestational age groups.

On the other hand, the prevalence reported in the current study is higher than that reported by Lu, Li [20] in Beijing, China (7%), Woldu, Teklehaimanot [7] in Ethiopia (7.2%) and Seale, Koech [8] in Kilifi (12%) [7, 8, 20]. The variations in the prevalence reported in the current study, and that reported by Cools, Jespers [16] and Seale, Koech [8] agrees with findings by Stoll and Schuchat [17] of occurrence of regional variation in GBS prevalence even among people sharing geographical boundaries and with similar socio-economic conditions [8, 16, 17]. Similarly, the difference in the prevalence could be explained by the culture method used in identification of GBS. This study used Granada agar in isolating GBS which has higher ability to isolate GBS compared to blood agar plates used by Lu, Li [20]. It has been noted that variation in GBS prevalence could be as a result of culture methods and type of medium used to isolate GBS [7].

The antimicrobial susceptibility pattern of isolated GBS showed high GBS resistance to penicillin G and ampicillin of 72.4 and 54.2% respectively. There was however low resistance to clindamycin and vancomycin at 30.4 and 24.1% respectively. Previous studies have reported GBS to be evenly susceptible to penicillin, ampicillin and cephalosporins [18, 21] even though bacteria with increasing minimum inhibitory concentration to penicillin and ampicillin have been reported [18, 21]. The high resistance of GBS to penicillin G reported in our study mirrors that reported by Mengist, Zewdie [22] of 77.3% [22]. Findings of the current study partly agrees with those of Lu, Li [20], Yoon, Jo [23] and Mengist, Zewdie [22] who reported a GBS resistance to clindamycin of 55.7, 55.4 and 50% respectively [20, 22, 23]. However, the resistance in the three studies is higher than that reported in the current study of 30.4%. Nonetheless, the isolates in Yoon, Jo [23] and Lu, Li [20] studies retained 100% susceptibility to penicillin, ampicillin and vancomycin. On the other hand, the resistance of GBS to clindamycin reported in our study is higher than that reported by Bolukaoto, Monyama [18] of 17.2%, even so, Bolukaoto, Monyama [18] isolates retained 100% susceptibility to penicillin, ampicillin, vancomycin and high level gentamycin even though they had high resistance to tetracycline of 86.7% [18].

Increase in resistance of GBS to β-lactam antibiotics has been attributed to alterations in the penicillin binding protein 2x [21] while resistance to clindamycin has been attributed to presence of erm-methylase gene [24]. In his study to determine antibiotic resistance of GBS among pregnant women in Garankuwa, South African, Bolukaoto, Monyama [18] reported methylation of *ermB* genes to be the single most common mechanism of resistance employed by isolated bacteria. Other mechanisms identified were efflux pump mediated by *mefA* genes and *ermTR* genes. This high resistance limits antibiotic use and restricts treatment to clindamycin and vancomycin.

All the known ten serotypes of GBS were found to be occurring in this study population. The most occurring serotype was Ia (75.9%) followed by III (62%), V (56%), VI (54%), Ib (53.7%), VII (53.5%), IX (53.3%), II (48%) and VIII (44.2%). Serotype IV was the least occurring at 36%. 66.7% of participants were found to harbour more than one serotype of GBS. These findings agree with Rench and Baker [25] who reported serotypes Ia, Ib, II and III as the most occurring, similarly, they mirror Lu, Li [20] who isolated eight GBS serotypes among pregnant women in his study with the exception of VII and IX. Nine GBS serotypes (Ia, Ib, II-VIII) have been reported to occur in Europe and USA [26]. Previous studies conducted in Kenya have reported the occurrence of six GBS serotypes Ia, III, V, VI, VII and VIII in a Mombasa cohort [16]. Our findings agree with observations of Dutra, Alves [27] who reported variations in the regional distribution and occurrence of GBS serotypes as this study found serotype Ia to be the most occurring in this population while Cools *et.al* reported serotype III as the most occurring among pregnant women in Mombasa [16, 27]. It is however, important to note that serotype distribution in a population could change with time. Yoon, Jo [23] in a GBS study conducted over a 20 years period found the dominant serotypes to change with time [23].

Serotype Ia (80.5%) and VI (81.5%) had significant resistance to penicillin G while serotypes Ia (61%), III (71%), IV (77.8%), VI (70.5), VII (65.25) and VIII (73.7%) registered significant resistant to ampicillin. Colonization with these serotypes was also a predictor of antimicrobial resistance with being colonized with serotype Ia predicting for penicillin G resistance (OR 4.8; CI: 1.265–18.311, *p* = 0.021) as was serotype VI (OR 3.38; CI:0.947–12.098, *p* = 0.061). Being colonized with serotypes III (OR 6.84; CI:1.899–24.672, *p* = 0.003), IV (OR 5.12; CI:1.372–19.077, *p* = 0.015), VI (OR 4.45; CI:1.353–14.653, *p* = 0.014), VII (OR 3.48; CI:0.99–12.242, *p* = 0.052) and VIII (OR 4.67; CI:1.255–17.358, *p* = 0.022) was a predictor for ampicillin resistance. Our findings differ from those by Yoon, Jo [23], who in their study, found no strains to be resistant to penicillin [23]. They also reported a resistance of 93.8% to clindamycin by serotype V. This level of resistance to clindamycin by serotype V is higher than that reported in our study of 22.7%.

Though some of the participants in this study reported having a history of risk factors associated with GBS such as stillbirths, abortions, ectopic pregnancy, history of preterm births, history of neonatal deaths, history of neonatal infection and history of fore water break more than 18 h to labour, no statistically significant association was found between these factors and GBS colonization in this study. Similarly, there was no association between maternal age, gestational age, parity and number of live birth with GBS colonization. Other studies have reported an association between GBS colonization and some of these risk factors. For examples Salat [9] had previously reported GBS colonization to be associated with a history of stillbirth in the same population as did Seale, Koech [8] in Kilifi and Doare, Jarju [5] in the Gambia who besides still birth, also noted GBS colonization to be associated with early onset neonatal disease and gestational age [5, 9]. Even though the current study did not find any association between GBS colonization and associated risk factors, the role of GBS in causing stillbirths and neonatal sepsis cannot be ruled out given the relatively high GBS prevalence, stillbirths and neonatal sepsis in this setting.

## Conclusion

The prevalence of Group B Streptococcus among pregnant women seeking antenatal services at KNH is high. Anorectal GBS colonization was higher in comparison to vaginal colonization. No significant association was found between GBS and stillbirths, abortions or ectopic pregnancy, history of preterm births, history of neonatal deaths, history of neonatal infection and history of fore water break more than 18 h to labour. Group B Streptococcus isolated in this study were resistant to all tested antimicrobials with the highest resistance being to penicillin G while the least resistance was to vancomycin. All known GBS serotypes were found occurring in this population with serotype Ia being the most prevalent. All serotypes had strains that were resistant to tested antimicrobials. There was a statistically significant association between isolation of serotypes Ia and VI and resistance to penicillin G while isolation of serotypes Ia, III, IV, VI, VII and VIII was associated with ampicillin resistance. None of the isolated serotypes was associated with either clindamycin or vancomycin resistance. This study suggests introduction of screening of pregnant mothers for GBS colonization followed by antimicrobial susceptibility testing on GBS positive samples to guide antibiotic prophylaxis. Future studies should look at how many of the GBS rectovaginally colonized pregnant women give birth to GBS infected children.

## Data Availability

The dataset used in this study is available from the corresponding author upon reasonable request and upon approval from the Kenyatta National Hospital-University of Nairobi Ethics Research Committee.

## Abbreviations

ANC: Antenatal care
CLSI: Clinical and Laboratory Standards Institute
EOD: Early-onset disease
GBS: Group B Streptococcus
GBSA: Group B Streptococcus Selective Agar
IQR: Interquartile range
KNH: Kenyatta National Hospital
OR: Odds Ratio

## References

[1] Mitima KT, Ntamako S, Birindwa AM, Mukanire N, Kivukuto JM, Tsongo K (2014). Prevalence of colonization by Streptococcus agalactiae among pregnant women in Bukavu, Democratic Republic of the Congo. J Infect Dev Ctries.

[2] Buchan BW, Olson WJ, Mackey TL, Ledeboer NA (2014). Clinical evaluation of the walk-away specimen processor and ESwab for recovery of Streptococcus agalactiae isolates in prenatal screening specimens. J Clin Microbiol.

[3] El Aila NA, Tency I, Claeys G, Saerens B, Cools P, Verstraelen H (2010). Comparison of different sampling techniques and of different culture methods for detection of group B Streptococcus carriage in pregnant women. BMC Infect Dis.

[4] Gilbert R (2004). Prenatal screening for group B streptococcal infection: gaps in the evidence. Int J Epidemiol.

[5] Doare KL, Jarju S, Darboe S, Warburton F, Gorringe A, Heath PT (2016). Risk factors for group B Streptococcus colonisation and disease in Gambian women and their infants. J Infect.

[6] Namavar Jahromi B, Poorarian S, Poorbarfehee S (2008). The prevalence and adverse effects of group B streptococcal colonization during pregnancy. Arch Iran Med.

[7] Woldu ZL, Teklehaimanot TG, Waji ST, Gebremariam MY (2014). The prevalence of group B Streptococus recto-vaginal colonization and antimicrobial susceptibility pattern in pregnant mothers at two hospitals of Addis Ababa. Ethiopia Reprod Health.

[8] Seale AC, Koech AC, Sheppard AE, Barsosio HC, Langat J, Anyango M (2016). Maternal colonisation with *Streptococcus agalactiae*, and associated stillbirth and neonatal disease in coastal Kenya. Nat Microbiol.

[9] Salat M (2009). Prevalence of GBS colonization in antenatal women at Kenyatta National Hospital [masters degree thesis].

[10] MoH, Health Mo (2016). Kenya reproductive, maternal, newborn, child and adolescent health (rmncah) investment framework.

[11] KNBS. Kenya National Bureau of Statistics, Ministry of Health, National AIDS Control Council, Kenya Medical Research Institute, National Council for Population and Development, The DHS Program II, editors. Kenya Demographic and Health Survey 2014. Nairobi: Government press. p. 2015.

[12] UNDP (2016). Sustainable Development Goals Knowledge Platform New York.

[13] KNHS (2015). Health records annual statistics.

[14] Jisuvei CS, Maina AN, Osoti A (2019). Prevalence, antimicrobial susceptibility patterns, serotypes and risk factors for group B Streptococcus rectovaginal isolates among pregnant women at Kenyatta National Hospital, Nairobi, Kenya. Trans R Soc Trop Med Hyg.

[15] Franklin R. Cockerill I, MD, Matthew A. Wikler M, MBA, FIDSA, Karen Bush P, Michael N. Dudley P, FIDSA, George M. Eliopoulos M, Dwight J. Hardy P, et al. Performance standards for antimicrobial susceptibility testing; twenty-first informational supplement. In: Institute CaLS, editor. Pennsylvania: Clinical and Laboratory Standards Institute 2011. p. 100–104.

[16] Cools P, Jespers V, Hardy L, Crucitti T, Delany-Moretlwe S, Mwaura M, et al. Multi-Country Cross-Sectional Study of Vaginal Carriage of Group B Streptococci (GBS) and Escherichia coli in Resource-Poor Settings: Prevalences and Risk Factors. PLoS ONE. 2016;11(1):e0148052. 10.1371/journal.pone.0148052. 10.1371/journal.pone.0148052 PMC4727807 26811897

[17] Stoll BJ, Schuchat A (1998). Maternal carriage of group B streptococci in developing countries. Pediatr Infect Dis J.

[18] Bolukaoto JY, Monyama CM, Chukwu MO, Lekala SM, Nchabeleng M, MRB M (2015). Antibiotic resistance of *Streptococcus agalactiae* isolated from pregnant women in Garankuwa, South Africa. BMC Res Notes.

[19] Boyer KM, Gadzala CA, Burd LI, Fisher DE, Paton JB, Gotoff SP (1983). Selective intrapartum chemoprophylaxis of neonatal group B streptococcal early-onset disease. I Epidemiologic rationale J Infect Dis.

[20] Lu B, Li D, Cui Y, Sui W, Huang L, Lu X (2014). Epidemiology of Group B streptococcus isolated from pregnant women in Beijing, China. Clin Microbiol Infect.

[21] Verani JR, McGee L, Schrag SJ (2010). Prevention of Perinatal Group B Streptococcal Disease Revised Guidelines from CDC.

[22] Mengist HM, Zewdie O, Belew A, Dabsu R (2017). Prevalence and drug susceptibility pattern of Group B Streptococci (GBS) among pregnant women attending antenatal care (ANC) in Nekemte Referral Hospital (NRH), Nekemte, Ethiopia. BMC Res Notes.

[23] Yoon IA, Jo DS, Cho EY, Choi EH, Lee HJ, Lee H (2015). Clinical significance of serotype V among infants with invasive group B streptococcal infections in South Korea. Int J Infect Dis.

[24] Metcalf BJ, Chochua S, Gertz RE, Hawkins PA, Ricaldi J, Li Z (2017). Short-read whole genome sequencing for determination of antimicrobial resistance mechanisms and capsular serotypes of current invasive *Streptococcus agalactiae* recovered in the USA. Clin Microbiol Infect.

[25] Rench MA, Baker CJ (1993). Neonatal sepsis caused by a new group B streptococcal serotype. J Pediatr.

[26] Ippolito D, James W, Tinnemore D, Huang R, Dehart M, Williams J (2010). Group B Streptococcus serotype prevalence in reproductive-age women at a tertiary care military medical center relative to global serotype distribution. BMC Infect Dis.

[27] Dutra VG, Alves VM, Olendzki AN, Dias CA, de Bastos AF, Santos GO (2014). Streptococcus agalactiae in Brazil: serotype distribution, virulence determinants and antimicrobial susceptibility. BMC Infect DisBMC Infect Dis..

